# Openness to Experience as a Moderator of the Relationship between Intelligence and Creative Thinking: A Study of Chinese Children in Urban and Rural Areas

**DOI:** 10.3389/fpsyg.2016.00641

**Published:** 2016-05-03

**Authors:** Baoguo Shi, David Y. Dai, Yongli Lu

**Affiliations:** ^1^Beijing Key Laboratory of Learning and Cognition and Department of Psychology, Capital Normal UniversityBeijing, China; ^2^ED233 Educational Psychology, University at Albany, State University of New York, AlbanyNY, USA; ^3^East China Normal UniversityShanghai, China; ^4^School of Psychology, Beijing Normal UniversityBeijing, China

**Keywords:** openness to experience, intelligence, creative thinking, moderating effect, children

## Abstract

Using testing and questionnaire methods, this study investigated the relationships among openness to experience, intelligence and creative thinking. This study focused on the moderating effects of openness to experience on the relationship between intelligence and creative thinking in a sample of 831 primary school students in China. The findings showed significant positive relationships among openness to experience, intelligence and creative thinking. In relation to the focus of this study, openness to experience moderated the relationship between intelligence and creative thinking. However, the correlation between openness to experience and creative thinking was stronger for urban children than for rural children, and the moderating effect existed only in urban settings.

## Introduction

Openness to experience refers to the extent to which a person actively seeks and appreciates different experiences and tolerates and explores novel situations (Pervin, 2002). It involves a host of related concepts, such as aesthetic experience, intellectual curiosity, and a preference for non-traditional or original things (Costa and McCrae, 1992; Ingram et al., 2013). Individuals with high level of openness to experience are interested in new things, especially new knowledge and art and unconventional ideas. They are usually characterized as adventurous, imaginative, knowledgeable, and creative. In contrast, people who are low in this variable are characterized as self-constrained, obedient, and adherent to established daily routines and procedures, and lacking in creativity. Within the Big Five personality dimensions, this factor is the one most closely related to creativity (Kaufman et al., 2015), and it has a compound label of Openness/Intellect (Ashton et al., 2000; Nusbaum and Silvia, 2011). According to Saucier (1992, 1994) and Johnson (1994), Openness and Intellect are two correlated but separable variables that represent equally key aspects of one broad factor, which is often called Openness/Intellect. DeYoung et al. (2007) adopted this perspective and proposed that Openness and Intellect can be viewed as different traits that are part of the organization of personality within the Big Five factors. Here, Openness means perceptual and aesthetic engagement, which is described by adjectives such as artistic, perceptive, poetic, and fantasy-prone. In contrast, Intellect means perceived intelligence and intellectual engagement, which is described by adjectives such as intellectual, intelligent, clever, and philosophical (DeYoung et al., 2014). Openness reflects personal differences in the exploration of perceptual or sensory information, whereas Intellect reflects personal differences in exploration through abstract information (Fayn et al., 2015). The common factor in Openness and Intellect is cognitive exploration, which involves seeking, detecting, comprehending and utilizing information (DeYoung et al., 2014). In the present study, we call this concept openness to experience.

Openness to experience is consistently associated with all measures of creativity (Kerr and McKay, 2013). Using methods of self-evaluation, peer rating and adjective checklists, McCrae and Ingraham (1987) found that openness to experience and divergent thinking were positively correlated with Gough’s creative personality test scores. King et al. (1996) replicated and extended McCrae and Ingraham’s (1987) research. They argued that the association of openness to experience and creativity was not only empirically supported but also theoretically meaningful. Openness to experience might not directly cause creativity, but it serves as a “catalyst” for the expression and exploration of creative ideas and activities. Feist (1998) conducted a meta-analysis to explore the relation between personality traits with scientific and artistic creativity. He found that high openness to new experience was a significant characteristic of creative people in both scientific and artistic domains. Batey et al. (2010) argued, based on their study of Big Five personality traits and ideational behavior (an indicator of creativity), that personality traits (especially openness to experience) predict creativity better than measures of cognitive ability. More specifically, some recent studies have examined the role of openness to experience in creativity. By using voxel-based morphometry, Li et al. (2014) found that openness to experience mediated the relation between the right posterior middle temporal gyrus (pMTG) volume and trait creativity (measured by the Williams creativity aptitude test). Kerr and McKay (2013) developed 1 general and 5 specific profiles of creative adolescents and found that high openness to experience was an important trait. Kaufman et al. (2015) used questionnaire methods with 1035 subjects and found that openness to experience (Openness/Intellect) had a significant effect on creativity. Specifically, Openness predicted creative achievement in the arts, whereas Intellect predicted creative achievement in the sciences.

In addition to its relation with creativity, openness to experience often shows positive associations with IQ test performance. For example, it correlated strongly with verbal intelligence (*r* = 0.44) in a sample of 335 adults (Schretlen et al., 2010) and showed a direct association with change in crystallized intelligence (Ziegler et al., 2015). This association is mainly because openness to experience reflects the expression of intelligence in the Big Five personality factors (McCrae and Costa, 1997). However, extensive research has been conducted on the relationship between intelligence and creativity (see Silvia, 2008 for a review). Overall, the literature is inconclusive on the issue (Sternberg and O’Hara, 1999; Jauk et al., 2013). For instance, Getzels and Jackson (1962) posited that intelligence and creativity are not correlated. Barron and Harrington (1981) posited that intelligence is correlated with creativity to some extent. A common perspective is that there might be a threshold effect; that is, intelligence and creativity are positively correlated to a point (i.e., below 120 points of IQ), but the correlation becomes trivial or non-existent above the threshold (Karwowski and Gralewski, 2013). This is why, overall, the size of the correlation is relatively modest (typically a correlation coefficient of approximately 0.17; Kim, 2005). Research with adolescents (Duan, 1998, 2000) supports the threshold hypothesis. In these studies, the high range of intelligence scores has a diminishing effect on a measure of creativity, suggesting that creativity does not entail very high intelligence. Russo (2004) compared the TTCT test scores of high IQ children with those of average IQ children and reached the same conclusion. However, Silvia (2008) cautioned against underestimating the relationship between intelligence as a higher-order factor and creativity. Silvia (2008) conducted a study with 226 college students and found that the predictive effect of intelligence diminished (from β = 0.43 to β = 0.26) when openness to experience was included in the regression equation for creativity.

The three-way relationship among intelligence, openness to experience, and creativity is a complex one that warrants scrutiny. Openness to experience may express intelligence or reflect creativity, or, as DeYoung et al. (2005) proposed, it may play the role of a type of motivated cognitive flexibility that is related to dopamine function, especially in the dorsolateral prefrontal cortex (DLPFC). Although Silvia’s (2008) study used openness to experience as a covariate or confounding variable, we consider it an essential piece of the puzzle. Creativity certainly requires cognitive abilities as measured by intelligence tests, but it also involves affective and cognitive processes to activate or invest the cognitive resources at one’s disposal (McCrae and Ingraham, 1987; Sternberg and Lubart, 1996; Dai and Sternberg, 2004; Dai and Shen, 2008). This view of creativity is consistent with Eysenck’s (1997) model of creativity, which features the interplay of cognitive ability (or intelligence), personality traits, and environmental factors. In line with Jay and Perkins (1997), we believe that (a) environmental factors can encourage a person’s creativity; (b) intelligence enables creativity, and cognitive abilities and processes are responsible for the production of novel and creative thoughts and thus can explain how creativity is materialized; and (c) personality traits such as openness to experience predispose individuals to novel and creative ideation and are thus an essential mechanism for explaining the extent to which cognitive resources are utilized to create novel ideas. Thus, the mechanisms of creativity involve the interplay of environmental, cognitive, and personality factors (Csikszentmihalyi, 1988; Csikszentmihalyi and Wolfe, 2000; Simonton, 2000). Given that disparities between urban and rural children in terms of intellectual stimulation and environmental resources (e.g., Sun and Xie, 2008; Li and Ranieri, 2013) are greater in China than in more developed countries, it is meaningful to ask whether systematic differences exist between urban and rural children with respect to the realization of creative potential which actually has been influenced by the interaction of cognitive variables and personality traits. In other words, we need to further explore how environmental factors (e.g., urban vs. rural) affect the role of openness to experience and intelligence in children’s creative ideation and performance.

King et al. (1996) examined the relations among the five-factor model of personality, verbal creative ability, and creative accomplishments. They found that creative ability shared a positive linear relation with accomplishments when openness to experience was at a high level. Individuals high in verbal creative ability but low in openness to experience reported relatively few creative accomplishments. They used the catalyst theory to explain the role openness to experience plays in this interaction. More recently, Ivcevic and Brackett (2015) used a questionnaire method with 223 high school students and found that openness to experience had a moderating effect on the relationship between emotional regulation ability and creativity. In another experimental study, van Tilburg et al. (2015) found that openness to experience emerged as a mediator in the relation between nostalgia and creativity. All of these results suggest that we need to consider the special role of openness to experience as a third variable in the study of creativity. Specifically, openness to experience may “activate” intellectual resources in certain environmental conditions, resulting in creative ideation and production. Therefore, the relationship between intelligence and creative ability is most likely moderated by openness to experience as a personality factor. In the psychological literature, the distinction between moderation and mediation is an important but subtle one (Baron and Kenny, 1986). Because openness to experience is a personality trait, it is better seen as a moderator that determines the presence and degree of the relationship between intelligence and creative thinking depending on the presence and degree of this trait. The current study tests the moderating effect in terms of the interaction effect of openness to experience and intelligence on creative thinking using a standard procedure (Baron and Kenny, 1986; Wen et al., 2005). Specifically, we hypothesize the following: (1) both openness to experience and intelligence are positively correlated with creative thinking; (2) there is a significant interaction effect between openness to experience and intelligence—that is, openness to experience moderates the effects of intelligence on creative thinking; and (3) there is a difference between urban and rural subjects in terms of the moderating effect of openness to experience.

## Materials and Methods

### Participants

Participants were 831 5th- and 6th-grade students, including 580 students from 4 urban schools and 251 students from 3 rural schools. The mean ages for 5th-graders and 6th-graders were 11.6 years old and 13.0 years old, respectively. The distribution of participants is presented in **Table 1**. The present research was approved by the Research Ethics Board of Capital Normal University. The parents and teachers of all participants were provided informed consent prior to engaging in the survey. Children were not asked to participate if they were unwilling or uncomfortable.

**Table 1:** Demographic distribution of participants.

	Urban school		Rural school		Total
	Male	Female	Male	Female	
5th Grade	174	132	47	60	413
6th Grade	148	126	67	77	418
Total	322	258	114	137	831

### Instruments

#### The Measure of Openness to Experience

The measure of *openness to experience* was derived from the *Five-Factor Personality Scale for Middle School Students* by Zhou et al. (2000), which assesses the “small five” personality factors of children and adolescents: openness to experience, extraversion, agreeableness, conscientiousness and neuroticism. This instrument was developed based on the NEO-PI-R (Costa and McCrae, 1992) and an interview study and included items such as “I have a rich imagination.” It showed good reliability (with a Cronbach’s α coefficient from 0.676–0.890) and concurrent validity (*r* = 0.593–749 with the NEO-PI-R) and was modified to examine the personality traits of elementary school children (Xiang et al., 2006). In the present study, only the 26-item openness to experience subscale (the correlation coefficient with Openness dimension of the NEO-PI-R is 0.659 according to the developer) was used. To ensure that the participants would fully understand the questionnaire, a pilot study was conducted with 12 5th-graders. As a result, some wording was adjusted for appropriate use with elementary school students. Participants rated how true each statement was about themselves on a five-point scale ranging from 1 = *not at all true* to 5 = *always true*. The scale consists of 5 sub-dimensions including insightfulness, innovativeness, brightness, exploration and imagination, and the total scores obtained ranged from 26 to 130.

The Cronbach’s α coefficient was 0.878 for the present study, and the split half coefficients were 0.779 and 0.782. Confirmatory factor analysis was performed for the 26-item openness subscale using the AMOS4.0 procedure. The results indicated good reliability and convergent validity, with χ^2^/df = 1.959, NFI = 0.971, IFI = 0.985, TLI = 0.983, CFI = 0.985, and RMSEA = 0.057.

#### The Measure of Intelligence

Intelligence was assessed by a Chinese version of Raven’s Standard Progressive Matrices (RSPM-CR), including 60 items. Raw scores were calculated to yield a total score, with a highest possible total score of 60. This test was revised by Zhang and Wang (1989) based on 5,108 Chinese participants with ages ranging from 5.5 to over 70. For this measure, the split-half reliability was 0.95, and the test–retest reliability was 0.82. The correlation between this test and the Chinese version of the Wechsler Intelligence Scale for Children- Revised (Lin and Zhang, 1986) was 0.71 (Zhang and Wang, 1989). In the current study, the Cronbach’s α coefficient of the SPM-CR was 0.68.

#### The Measure of Creativity

Children’s creativity was measured by the Test of Divergent Thinking. The test contains four items; both verbal and non-verbal tasks were included. Three items were selected from the Torrance Tests of Creative Thinking (TTCT) (Torrance, 1966, 1993): the elf task (verbal), the parallel lines task (non-verbal), and the unfinished drawings task (non-verbal). The elf task requires subjects to ask questions as many as possible about one picture on which an elf appears. In the parallel lines task, participants were asked to draw pictures as many as possible based on 30 pairs of parallel lines by adding lines on each figure. Similarly, the unfinished drawings task also requires participants to draw pictures based on 10 incomplete figures. These tasks are associated with adequate evidence of reliability and validity (Kaufman et al., 2008). The fourth question (verbal) was developed by the researchers with reference to related creativity tests (Shi et al., 2012). It was used in the pilot study of the project titled “Cross-Cultural Comparison of Scientific Creativity between Eastern and Western Youths” among 127 7th-grade students and showed high discrimination among subjects, suggesting viability for research use. Specifically, the task states, “A scientist found an object in a scientific expedition. Please use your imagination and guess what it might be. Try to provide as many original ideas as possible.” The presented object is a vague, abstract drawing that could be interpreted in numerous ways. The rationale for creating the item was based on enhancing its construct validity (including both verbal and graphic tasks) and reducing the testing time (Shi et al., 2012).

The duration of this creativity test was approximately 30 min. Reliability analysis revealed that the Cronbach’s α coefficient for the composite measure was 0.74. Two raters individually scored the four items for 50 participants, and the inter-rater reliabilities (Pearson product moment correlation) ranged from 0.88 to 0.92.

According to the definition of creative thinking, based on the scoring criteria of TTCT and Hu et al. (2004), each item was individually rated on fluency, flexibility and originality after checking its appropriateness. Specifically, *fluency* refers to the effective number of responses the test taker gives to a divergent thinking question. *Flexibility* refers to the numbers of categories of participants’ responses. *Originality* refers to the uniqueness of a response in terms of the percentage of participants who gave the same response. If the percentage is below 5%, the participant receives two points. If the percentage is 5–10%, one point is given. If the percentage is higher than 10%, the score is zero (no points received). Total scores were obtained by summing the individual scores on all test items on fluency, flexibility and originality. Finally, three total scores were transformed to standardized scores and summed to yield the total score for creative thinking.

### Procedure

In each classroom, participants were administered two paper-pencil tests: the creative thinking test followed by the openness to experience questionnaire and the intelligence test. The participants completed background information and read the instructions under the guidance of test administrators before taking the test. The actual testing began when all the participants understood the requirements. The questionnaires and tests were collected as soon as they were completed. It took approximately 2 class periods (80 min) to complete all the tasks involved.

## Results

### Preliminary Analyses

**Table 2** presented the means and standard deviations across school type, grade and gender for the study variables.

**Table 2:** Means (*M*) and standard deviations (*SD*) across school type, grade and gender for the study variables.

Variables	Urban school	Rural school	5th Grade	6th Grade	Male	Female	Total
	(*n* = 580)	(*n* = 251)	(*n* = 413)	(*n* = 418)	(*n* = 436)	(*n* = 395)	(*N* = 831)
Openness	91.507 (15.240)	88.928 (13.259)	90.475 (15.030)	90.979 (14.400)	89.477 (14.639)	92.109 (14.682)	90.728 (14.710)
Intelligence	42.693 (8.129)	36.904 (7.891)	39.484 (8.447)	42.388 (8.274)	41.440 (8.170)	40.397 (8.789)	40.945 (8.481)
Creative Thinking	0.861 (2.981)	-1.988 (1.036)	-0.345 (2.434)	0.340 (3.210)	-0.131 (2.701)	0.144 (3.042)	0.000 (2.870)
Verbal task	1.179 (5.141)	-2.725 (2.232)	-0.261 (4.522)	0.258 (5.074)	-0.304 (4.805)	0.336 (4.802)	0.000 (4.812)
Non-verbal task	1.628 (5.169)	-3.762 (2.120)	-0.736 (4.334)	0.728 (5.690)	-0.108 (4.714)	0.119 (5.520)	0.000 (5.111)

*Creative thinking, verbal and non-verbal tasks score are the sum of standardized score of fluency, flexibility and originality.*

A multivariate analysis of variance (MANOVA) was performed to examine the impact of demographic variables on the study measures. The results showed a significant main effect of school type [Wilks’ lambda = 0.713, *F*_(5.819)_ = 65.833, *p* < 0.001, $\eta_{p}^{2}$ = 0.287] on all of these measures, a significant main effect of grade [Wilks’ lambda = 0.957, *F*_(5.819)_ = 7.301, *p* < 0.001, $\eta_{p}^{2}$ = 0.043] on intelligence and creative thinking, a significant main effect of gender [Wilks’ lambda = 0.985, *F*_(5.819)_ = 2.464, *p* < 0.05, $\eta_{p}^{2}$ = 0.015] on openness and creative thinking, and a significant school type × grade interaction [Wilks’ lambda = 0.957, *F*_(5.819)_ = 7.360, *p* < 0.001, $\eta_{p}^{2}$ = 0.043] on intelligence and creative thinking. Further analysis indicated urban children scored higher than rural children on all of these measures, Grade 6 scored higher than Grade 5 on intelligence and creative thinking, and girls scored higher than boys on openness and creative thinking. These findings indicate the demographic variables must be employed as control variables in the later correlation and regression analyses.

The correlation matrix of the openness to experience, intelligence and creative thinking variables are shown in **Table 3**.

**Table 3:** Partial correlation analysis of openness, intelligence and creative thinking (controlling for school type, grade and gender).

	1	2	3	4	5	6	7
(1) Openness	–						
(2) Intelligence	0.172^∗∗^	–					
(3) Creative thinking	0.374^∗∗∗^	0.306^∗∗∗^	–				
(4) Fluency	0.363^∗∗∗^	0.294^∗∗∗^	0.978^∗∗∗^	–			
(5) Flexibility	0.342^∗∗∗^	0.321^∗∗∗^	0.942^∗∗∗^	0.930^∗∗∗^	–		
(6) Originality	0.356^∗∗∗^	0.254^∗∗∗^	0.915^∗∗∗^	0.843^∗∗∗^	0.745^∗∗∗^	–	
(7) Verbal task	0.329^∗∗∗^	0.252^∗∗^	0.895^∗∗∗^	0.888^∗∗∗^	0.837^∗∗∗^	0.813^∗∗∗^	–
(8) Non-verbal task	0.340^∗∗∗^	0.303^∗∗∗^	0.913^∗∗∗^	0.875^∗∗∗^	0.863^∗∗∗^	0.850^∗∗∗^	0.655^∗∗∗^

*N = 826–831; ^∗∗∗^*p* < 0.001, ^∗∗^*p* < 0.01.*

As the results found, after controlling for the effects of school type, grade and gender, openness, intelligence, creative thinking were significantly correlated with one another. Because the intercorrelations of the three subtests of creativity (fluency, flexibility, and originality) were sizable (ranging from 0.745 to 0.930) and were close or equal to the reliabilities of these subtests, for the difference statistics and the ensuing analysis, no fine discrimination of these constructs could be empirically justified. Instead, only the composite score of creativity and verbal vs. non-verbal subtests were used to index creativity.

Moreover, as an estimate of the unique contributions of openness above and beyond the contributions of intelligence to the variance in creative thinking, the independent contributions of openness were examined. When the effects of intelligence were statistically partialled out, the correlation coefficients of openness and the three measures of creativity were 0.343 (for the total score), 0.299 (for the verbal tasks) and 0.307 (for the non-verbal tasks), respectively. Incidentally, when the effects of openness were partialled out, the correlations between intelligence and creativity were 0.265, 0.210 and 0.264, respectively. Taken together, these results support the first research hypothesis that openness and intelligence have independent contributions to the variance of creative thinking.

### Using Hierarchical Regression Analysis to Test the Moderation Hypothesis: Interaction Effects of Openness to Experience and Intelligence on Creative Thinking

At the center of the present study is the interaction effect of openness to experience and intelligence on creative thinking. Baron and Kenny (1986) and Wen et al. (2005) suggested that when both the independent variables and the moderator were continuous variables, the interaction effects could be assessed by adding the centralized product of the independent and moderator variables to the hierarchical regression analysis. Therefore, based on the previous correlation analyses, a hierarchical regression analysis was conducted to test the interaction effects of openness to experience and intelligence on the scores of creative thinking. In Step 1, school type, grade and gender (transformed into dummy variables) were entered to control for the possible effects of these covariates. Then, openness to experience and intelligence were entered in Step 2 to test their main effects on creative thinking. Finally, the openness to experience by intelligence interaction term (after being decentralized) was entered in Step 3 to estimate the interaction effects (i.e., the moderating effect of openness to experience). The results are presented in **Table 4**.

**Table 4:** Hierarchical regression analysis summary for openness and intelligence predicting creative thinking.

	Creative thinking (Composite)			Verbal task			Non-verbal task		
	β	Δ*R*^2^	*R*^2^	β	Δ*R*^2^	*R*^2^	β	Δ*R*^2^	*R*^2^
Step 1		0.242	0.242		0.157	0.157		0.275	0.275
School type									
Grade									
Gender									
Step 2		0.152	0.394		0.124	0.281		0.128	0.403
Openness	0.269^∗∗∗^			0.257^∗∗∗^			0.232^∗∗∗^		
Intelligence	0.239^∗∗∗^			0.203^∗∗∗^			0.236^∗∗∗^		
Step 3		0.021	0.415		0.010	0.292		0.024	0.427
O×I	0.146^∗∗∗^			0.103^∗∗∗^			0.157^∗∗∗^		
			
	*F*(6,824) = 97.427^∗∗∗^			*F*(6,824) = 56.528^∗∗∗^			*F*(6,824) = 102.425^∗∗∗^		

*β, standardized regression coefficient; O, openness, I, intelligence. ^∗∗∗^*p* < 0.001.*

The results of the hierarchical regression analysis indicate that after controlling for school type, grade and gender, openness to experience and intelligence significantly predicted the composite measure of creative thinking; the standardized regression coefficient β was 0.269 (*t* = 9.775, *p* < 0.001) and.239 (*t* = 8.184, *p* < 0.001), respectively. The interaction term, when entered in Step 3, also predicted creative thinking; the standardized regression coefficients were β *=* 0.146 (*t* = 5.408, *p* < 0.001), Δ*R*^2^= 0.021 [Δ*F*_(1,824)_ = 29.251, *p* < 0.001], which indicates that there was a significant interaction effect between openness to experience and intelligence. The regression model accounts for a total of 41.5% of the variance of the measure. Moreover, the interaction effects on the verbal and non-verbal tasks were statistically significant after controlling for the main effects of openness to experience and intelligence: for the verbal task, β = 103 (*t* = 3.478, *p* < 0.01), Δ*R*^2^ = 0.010 [Δ*F*_(1,824)_ = 12.097, *p* < 0.001], and for the non-verbal task, β = 0.157 (*t* = 5.888, *p* < 0.001), Δ*R*^2^ = 0.024 [Δ*F*_(1,824)_ = 34.671, *p* < 0.001]. These results support our second hypothesis that openness to experience moderates the relationship between intelligence and creative thinking.

### Plotting the Interaction Effects

**FIGURE 1:**
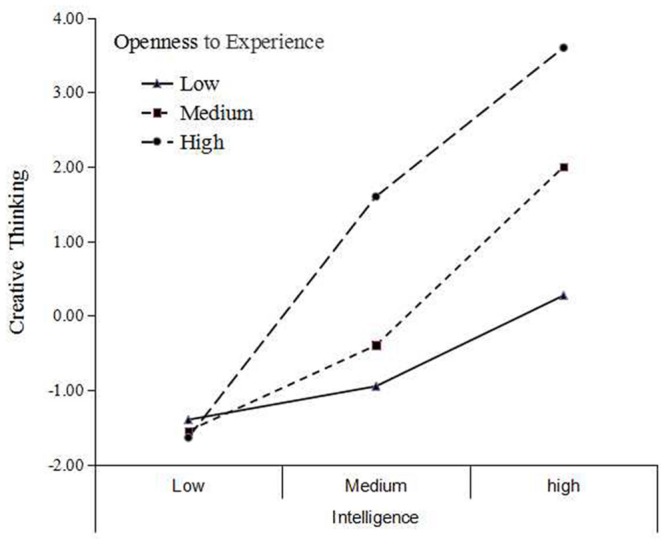
**Interaction effect of openness and intelligence on the composite measure of creative thinking**.

To more thoroughly discuss the interaction effects of openness to experience and intelligence as well as the moderating effect of openness to experience on the relationship between intelligence and creative thinking, we followed prior studies (King et al., 1996; Ivcevic and Brackett, 2015) and grouped the participants into a *high intelligence group* (scored one standard deviation above the mean score of intelligence), a *medium intelligence group* (scored within the range of one standard deviation above and below the mean), and a *low intelligence group* (scored one standard deviation below the mean). The same procedure was applied to the openness to experience score. **Figure 1** illustrates the interaction effects of openness to experience and intelligence on the composite measure of creative thinking.

As shown in **Figure 1**, the relationship between intelligence and creative thinking is consistently strongest for the high openness to experience group (steeper lines) and is weakest for the low openness to experience group, with the median group in between. For example, **Figure 1** reveals a notable interaction effect of openness to experience and intelligence on the composite measure of creative thinking. Those participants who scored low in openness to experience, regardless of their intelligence scores, generally achieved the lowest scores for creative thinking. For participants with medium or high scores in openness to experience, intelligence levels better predicted creativity.

### Exploratory Analyses of Urban and Rural Children: The Role of Openness to Experience on Creativity

Given the nature of the sample for this study, we further explored whether environmental factors may affect the role of openness to experience in children’s creative ideation and performance. This sample included 580 urban children and 251 rural children. The mean comparisons show that urban children scored slightly higher than rural children on openness to experience (91.51 vs. 88.93, *t* = 2.33, *p* < 0.05) and intelligence (42.69 vs. 36.90, *t* = 9.51, *p* < 0.001). However, the differences between the two groups on composite creativity measures (summed from standardized scores of fluency, flexibility and originality) were more distinct (0.86 vs. -1.99, *t* = 14.76, *p* < 0.001). To account for the differences, we compared the correlation coefficient between openness to experience and creative thinking of urban children with that of rural children. For urban children, after controlling for grade and gender, the correlation coefficient of openness to experience and the total score of creative thinking was 0.422 (*p* < 0.001); the correlations between openness to experience and the verbal and non-verbal creativity tasks were 0.375 (*p* <0.001) and 0.383 (*p* < 0.001), respectively. For rural children, after controlling for grade and gender, the correlation coefficient of openness to experience and the total score of creative thinking was.220 (*p* < 0.001); the correlations between openness to experience and the verbal and non-verbal creativity tasks were 0.151 (*p* < 0.05) and 0.192 (*p* < 0.01), respectively.

**FIGURE 2:**
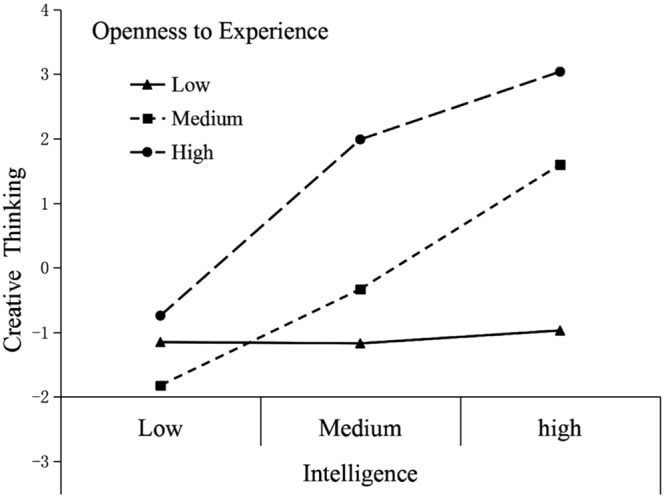
**Interaction effect of openness and intelligence on creative thinking with urban children**.

**FIGURE 3:**
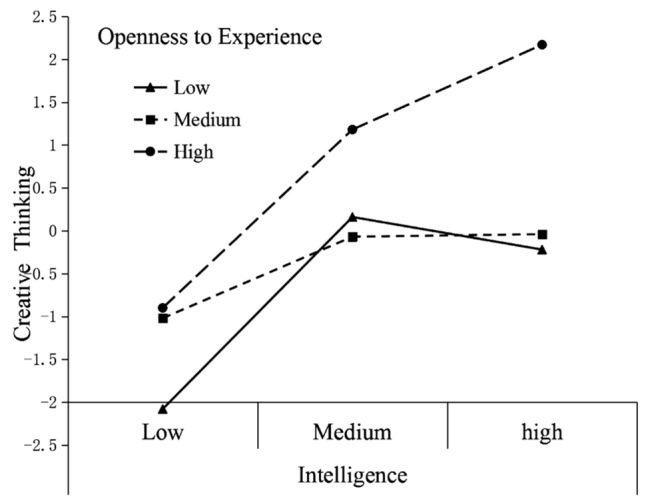
**Interaction effect of openness and intelligence on creative thinking with rural children**.

A Fisher z transformation was performed to compare these correlations across groups. The results of one-tailed tests revealed that for the correlation coefficient of openness to experience and the composite score of creative thinking, the correlation was significantly higher for the urban children than for rural children (*Z* = 2.963, *p* < 0.01). The results showed that the relationship of openness to experience and creative thinking was stronger for urban children than for rural children. Furthermore, according to the statistical method used above, two hierarchical regression analyses were conducted to test the interaction effects of openness to experience and intelligence on creative thinking for urban and rural students. Given the overlap among different measures, we use only the composite measure of creative thinking. The results showed that for urban subjects, openness to experience (β = 0.311, *t* = 8.155, *p* < 0.001) and intelligence (β = 0.248, *t* = 6.596, *p* < 0.001) significantly predicted the composite measure of creative thinking. Their interaction also predicted creative thinking [β = 0.143, *t* = 3.861, *p* < 0.001, Δ*R*^2^= 0.018, Δ*F*_(1,574)_ = 14.910, *p* < 0.001], which means that openness to experience significantly moderated the relation between intelligence and creativity in urban settings (**Figure 2**). More specifically, the association between intelligence and creative thinking is strong for both the high and median openness to experience group (steeper slopes). However, those urban participants who scored low in openness to experience achieved the lowest scores for creative thinking regardless of their intelligence level. For rural children, although openness to experience (β = 0.222, *t* = 3.361, *p* = 0.001) and intelligence (β = 0.233, *t* = 3.809, *p* < 0.001) significantly predicted the composite measure of creative thinking, their interaction did not predict creative thinking [β = 0.030, *t* = 0.450, *p* > 0.05, Δ*R*^2^= 0.001, Δ*F*_(1,245)_ = 0.202, *p* > 0.05], which means that the moderating effect of openness to experience does not exist for rural subjects (**Figure 3**).

## Discussion

Openness to experience and intelligence are two personality and cognitive constructs used in the literature to explain major factors that influence individual creativity. Although the point that personality traits are different from cognitive abilities has been challenged (DeYoung, 2011), the distinction between openness to experience, measured by self-report questionnaires, and intelligence, assessed by standardized psychometric tests, is clear. The self-report questionnaire method reflects the subjective perception of one’s behavioral tendencies, which may include potential, whereas standardized psychometric tests reflect the objective level of one’s actual ability (Zhang, 2003), such as intelligence and creative ability. Given the coefficient (*r* = 0.30) between Openness/Intellect and intelligence tests found by prior studies (Ackerman and Heggestad, 1997; DeYoung, 2011), these variables cannot be viewed as homogenous. According to prior studies (DeYoung et al., 2007, 2014), Openness and Intellect are two subtraits that constitute a broader aspect. When we employ the questionnaire of openness to experience, it includes content reflecting both Openness and Intellect. Based on this knowledge, the significant positive relationship between openness to experience and intelligence in the current study is understandable. Based on similar research (e.g., McCrae and Ingraham, 1987; King et al., 1996; Duan, 1998, 2000; Russo, 2004; Jauk et al., 2013; Karwowski and Gralewski, 2013; Kerr and McKay, 2013; Li et al., 2014; Kaufman et al., 2015; van Tilburg et al., 2015), the present study lends further support to the argument that openness to experience makes an independent contribution to the variance of creativity. A unique contribution of the present study is the exploration of the moderating effect of openness to experience on the relationship between intelligence and creative thinking; the strength of the relationship between the two is contingent on the degree or level of openness to experience. According to Ziegler et al. (2012, 2015), the effect of fluid intelligence (Gf) on immediate performance (Gc) decreases along with the increase of openness, which suggests a possible interaction between Gf and openness. Based on this knowledge, we formulate the hypothesis that openness might play a moderator role between intelligence and creative thinking. The findings of this study support the hypothesis, and provide new evidence for McCrae and Ingraham’s (1987) conception of openness to experience as a “catalyst” and King et al.’s (1996) and Ivcevic and Brackett’s (2015) hypotheses that openness to experience plays a special role as a third variable in the study of creativity. More specifically, the relationship between intelligence and creative ability in the current study is moderated by openness to experience as a personality factor. Intelligence can predict creative thinking only when the openness to experience score is medium or high; conversely, when the openness to experience level is low, intelligence exerts a very limited influence on creative thinking. Although statistically intelligence can also be considered as a moderator of the relationship between openness to experience and creativity, it is theoretically more justifiable to treat openness to experience as a moderator, as intelligence is better seen as an indicator of ability or aptitude, which is realized to various degrees depending on other certain conditions (i.e., moderation), openness to experience being one of them. In other words, the exercise of intelligence in generating creative ideas and solutions is contingent on the facilitative role of openness to experience, not the other way around.

Although several recent studies did reveal an interaction between intelligence and openness to experience, the effect is different with the current study. For example, Ziegler et al. (2012, 2015) found a buffering effect of openness to experience in the relationship between fluid intelligence and crystallized intelligence, suggesting that the correlation between Gf and Gc would decrease with an increase in openness to experience. Similarly, Zhang and Ziegler (2015) found another buffering effect of openness to experience in the association between fluid intelligence and secondary school students’ scholastic performance. In contrast, the current study found an independently enhancing effect of openness, though it does reveal the interaction between intelligence and openness to experience, indicating that once an individual score above a specific level on openness to experience, intelligence will have a stronger influence on creative thinking. The difference between both effects may be related to the nature of creativity. As we know, creativity is a special construct which different with Gc and scholastic performance greatly in terms of many aspects. Specifically, the current study uses divergent thinking test as an indicator of creativity. For such test, there is no a standard answer to the question. However, this situation does not exist for the intelligence test or for scholastic test. According to Ziegler et al. (2012), people with high openness to experiences are more likely to engage in a new and challenging environment and to acquire novel knowledge, thus a student with high intelligence and strong curiosity and imagination may perform better on the generation of creative responses. But it may do not work when this student take a conventional exam in a school context, since such characteristics might bring low interest and distraction in what the teacher taught in classroom (Zhang and Ziegler, 2015). Future studies should verify these ideas further.

The findings that moderation effect of openness to experience is more distinct in urban than rural children is worth discussing in terms of how the dynamics of intelligence and openness to experience works in the development of creativity. Based on the “catalyst” theory, they seem to suggest that, everything else being equal, a presumably more intellectually stimulating environment in urban settings may make children with high openness to experience more active in seeking intellectual opportunities and thus more likely to “convert” their intellectual energy into creative use. This interpretation, though tentative, is consistent with Eysenck’s (1997) theory of the interplay of environmental, personal, and cognitive factors. Moreover, the moderating effect of openness to experience was found only for urban subjects, which may indicate that the relationship among personality and cognitive ability depends on the level of these variables considering the distinction of the scores of both openness to experience and intelligence between urban and rural children. A possible explanation is that there might be a threshold effect for openness to experience, just as there is for intelligence (Guilford and Christensen, 1973), when correlated with creativity. Only a higher level of openness to experience can lead to a positive correlation with creativity; however, it becomes trivial or non-existent when the threshold is lower. Future studies need to verify this hypothesis.

Because the participants in the present study were 5th- and 6th-grade elementary school children, it is worthwhile to consider the extent to which their openness to experience scores truly reflect their tendency to seek, tolerate, and appreciate novel experiences. Psychologically speaking, an individual’s personality is not fully developed and stabilized until adulthood. However, the findings of the present study suggest that it is meaningful to study the personality functioning of preadolescents. In fact, a considerable amount of recent research has been conducted on the assessment, types, and developmental stability of elementary school children’s personality (e.g., Laidra et al., 2007; Edmonds et al., 2013; Neuenschwander et al., 2013). The results from extensive cross-cultural studies on creativity (e.g., Ye et al., 1988; Rudowicz et al., 1995; Ng, 2003; Hu et al., 2004; Niu et al., 2007) have documented the disparity in creativity between Chinese adolescents and their counterparts in developed countries. One possible reason is a lack of opportunity to cultivate creativity through openness to experience during childhood and adolescence. The findings of the present study on preadolescents have developmental and educational implications for childhood. More research is needed to test the openness to experience moderation hypothesis in developmental contexts (e.g., college students) to clarify the role of personality and intelligence in creativity development.

In closing, the finding of this study that openness to experience tends to enhance the role of intelligence in creative ideation and performance, coupled with the evidence of a stronger relationship between openness to experience and creativity for urban children compared to rural children, suggests a complex interplay of environmental, cognitive, and personality factors in creative thinking and creativity development. Future research is warranted to further clarify this interplay and to develop a more integrated theory of intelligence, personality, and creativity. Consider the facts that the Standard Progressive Matrices mainly assesses one’s fluid intelligence, and openness to experience is measured at the domain level but not at the facet level in the current study, measures which are more robust and more comprehensive might be adopted in the future. Moreover, because the theory and practice of Openness/Intellect (Ashton et al., 2000; DeYoung et al., 2005, 2007, 2014; Nusbaum and Silvia, 2011; Kaufman et al., 2015) in relation to openness to experience has emerged in recent years, the possible roles of Openness and Intellect in creativity need to be examined.

## Author Contributions

BS and YL designed research; BS carried out research; BS and DD analyzed data results; BS, DD, and YL wrote the manuscript.

## Conflict of Interest Statement

The authors declare that the research was conducted in the absence of any commercial or financial relationships that could be construed as a potential conflict of interest.
